# Assessment of laying-bird welfare following acaricidal treatment of a commercial flock naturally infested with the poultry red mite *(Dermanyssus gallinae)*

**DOI:** 10.1371/journal.pone.0241608

**Published:** 2020-11-19

**Authors:** Déborah Temple, Xavier Manteca, Damián Escribano, Marina Salas, Eva Mainau, Eva Zschiesche, Ivo Petersen, Roser Dolz, Emmanuel Thomas

**Affiliations:** 1 School of Veterinary Medicine, Universitat Autonoma de Barcelona, Bellaterra, Barcelona, Spain; 2 MSD Animal Health GmbH, Zur Propstei, Schwabenheim, Germany; 3 MSD Animal Health, Calle de Josepha de Valcárcel, Madrid, Spain; Beni Suef University, Faculty of Veterinary Medicine, EGYPT

## Abstract

The poultry red mite (PRM), *Dermanyssus gallinae*, a potential vector of pathogens to animals and humans, causes impaired bird welfare. A study investigated changes in behavioural variables, physiological biomarkers, and health parameters following acaricidal treatment of PRM infestation of laying hens on a commercial farm. Mite traps determined the challenge to 12,700 hens before and after drinking water administration of the acaricide, fluralaner (Exzolt^®^, 0.5 mg/kg; Weeks 0 and 1). Weekly daytime direct observations and night-time video recordings monitored bird behaviours from Weeks -6 through +6. Blood samples were collected from randomly-selected birds (Weeks -6, -1, and +6). Following treatment, mite count reductions (>99%) were statistically significant (*P* < 0.0001), as were night-time reductions in the percent of hens showing activity, preening, head scratching (all *P* < 0.0001), and head shaking (*P* = 0.0007). Significant daytime reductions were observed in preening and head scratching (both *P* < 0.0001), head shaking (*P* = 0.0389), severe feather pecking (*P* = 0.0002), and aggressive behaviour (*P* = 0.0165). Post-treatment, comb wounds were significantly reduced (*P* = 0.0127), and comb colour was significantly improved (*P* < 0.0001). Heterophil/lymphocyte ratio was significantly reduced at Weeks 1 and 6 (*P* = 0.0009 and *P* < 0.0001, respectively). At Week 6, blood corticosterone (*P* = 0.0041) and total oxidant status (*P* < 0.0001) were significantly reduced, and haemoglobin and mean corpuscular haemoglobin significantly increased (*P* < 0.0001). Farm production records indicated that those post-treatment improvements were accompanied by significant reductions in weekly mortality rate (*P* = 0.0169), and significant recovery in mean weekly egg weights (*P* < 0.0001) and laying rate (P < 0.0001). The improvements in behavioural variables, physiological biomarkers, and health parameters that were observed following the elimination of PRM on a commercial farm indicate that infestations can be a cause of reduced hen welfare.

## Introduction

The poultry red mite (PRM), *Dermanyssus gallinae* affects all poultry production types, from backyard and organic farms to more intensive, enriched cage or barn systems [1]. The PRM is a vector of viral and bacterial pathogens to animals and humans, and may also predispose birds to clinical and subclinical diseases [1, 2]. Zoonotic infections and human contact dermatitis can be a consequence of living or working in close association with infested poultry [3]. Moderate to severe infestation of commercial laying flocks can increase food consumption, reduce egg production and quality, and may lead to increased mortality [4–7].

In laying hen facilities *D*. *gallinae* hide in cracks and crevices, close to hen resting places, clustered in pheromone-induced aggregations, where they digest the recently taken blood meal and reproduce, emerging every few days, usually during darkness, to feed for 30 to 60 minutes [8–13]. Despite the abundant literature on this parasite, little is known about the degree to which infestations impair the welfare of the laying hen host, although two reports indicate that poultry well-being is likely to be adversely impacted by infestation. In one report, Kilpinen et al. observed behavioural changes of increased grooming behaviour in mite-infested hens relative to uninfested controls [14]. In another report, measurements of corticosterone, adrenaline, noradrenaline, albumin and α-, β- and γ- globulins demonstrated that heavy infestation with PRM stimulated the hypothalamic-pituitary-adrenal (HPA) cortex axis and the sympatho-adrenomedullar system [15]. A corollary of those findings is that mite elimination would improve hen welfare. To test this hypothesis a study was undertaken under commercial production conditions to assess changes on a wide range of animal welfare indicators following acaricidal treatment of hens on a naturally mite-infested commercial building. The selected acaricide was fluralaner, a systemically acting compound of the isoxazoline class that has been shown to eliminate PRM infestations from hens and poultry houses [16].

## Methods

The objective of the study was to evaluate changes in behavioural and haematological parameters, blood stress biomarkers, and general hen health following acaricidal treatment of hens in a PRM-infested poultry unit. The impact of these treatments on production parameters was also estimated.

The study was conducted in alignment with the principles of the Good Clinical Practice VICH GL9 (GCP) (CVMP/VICH/595/98-Final) [17] on a commercial egg production farm and was approved by the Ethical Committee on Animal and Human Experimentation of the Universitat Autonoma de Barcelona. Before the beginning of the experiment, an informed consent was signed by the farm’s owner, and owner agreement was obtained that any non-study poultry on the farm would also be treated to avoid the risk of mite cross-contamination.

The family-owned farm was selected because it was appropriate for completing a GCP-adherent study and had a history of infestation with the PRM. A pre-requisite for farm selection was having conditions that would allow assessment of PRM infestation and monitoring of hen behaviours. Suitable equipment (medication tank) was available to ensure accurate delivery of fluralaner via drinking water, using nipple drinkers with drip cups. The house selected for the study was a commercial building, 12 by 80 m, containing 12,700 healthy layer ISA Brown hens in Zucami furnished cages (Zucami Poultry Equipment, SLU) (55 hens/cage). At the start of the study (Week—6) the birds were approximately 29 weeks of age and entered the building at 18 weeks of age. Throughout the study, birds were fed a commercial ration (Nanta, SA) containing 16.4% crude protein, 3.9% calcium and 0.5% available phosphorus. Feed was provided according to the farm’s standard procedure. The temperature inside the building ranged between 16 and 22°C (measures made between 10 am and 11am three times a week).

Any treatment against *D*. *gallinae*, by oral administration, spray application, or treatment of premises in the 14 weeks before study start was not performed. The only restriction during the study was the application or administration of any non-study acaricidal treatment. Routine health interventions (e.g., vaccination, vitamin supplementation…) and medical care were performed but were documented. General health observations of the birds were completed by the veterinary investigator at regular intervals.

### Treatment

A commercially available solution of fluralaner provided for use in drinking water (10 mg /mL, Exzolt^®^, MSD Animal Health) was administered twice, each calibrated to achieve a dose rate of 0.5 mg of fluralaner per kg body weight, separated by a one-week interval [18]. The volume of water consumption by birds in the house was estimated on the day before administration and the required volume to be medicated was determined by the average weight of a representative sample of 50 birds times the number of birds (12,700) to be treated. The day of each administration, medicated water was freshly prepared and provided to the birds. Sufficient time was allowed to ensure that all birds would receive the required dose (treatment duration on Day 0 was 6h 26 mn, and 7h 6 mn on Day 7). Water was provided *ad libitum*. No other source of drinking water was available during the medication period.

### Monitoring of poultry red mite infestation

To provide insight into the environmental PRM burden, 20 individually labelled traps (Avivet^®^) were evenly installed among different rows throughout the house, avoiding any site near an air inlet, following the standard operating procedure for houses containing fewer than 25,000 birds, including trap placement on perches and avoidance of mite clusters [19, 20]. Traps were placed at weekly intervals, beginning six weeks prior to the first fluralaner administration, at three days following the first administration (Week 0, Day 3), then two days following the second administration (Day 9, Week 1), then on Day 14 (Week 2) and subsequently at weekly intervals through Week 6. Two days after placement, each trap was collected, identified by its position and date, and sealed individually in a small plastic bag that was placed into a larger plastic bag. Samples were then stored in a freezer at −18 to −20°C until the end of the study and shipped on dry ice to a laboratory where mites in each trap, and in the plastic bag containing the trap, were poured into a petri dish. The cardboard of the trap and the plastic bag that had contained the trap were carefully examined for remaining mites and any that were found were added to the mites in the dish. For traps containing up to 250 mg of *D*. *gallinae* (total weight of eggs and mobile stages), all mobile stages of *D*. *gallinae* were counted; for traps containing more than 250 mg, a subsample of approximately 100 mg was used.

### Assessment of poultry red mite effects on infested hens

#### Behavioural observations

Weekly assessments of bird behaviour were performed from Week -6 through Week 6 during daytime by direct observation, and during night-time by video recording. In Weeks 0 and 1, these observations were completed three and four days following the first and second fluralaner administrations, respectively (Table 1).

**Table 1:** General schedule of study activities.

Data collected	Units	Study week												
		-6	-5	-4	-3	-2	-1	0	1	2	3	4	5	6
Mite traps^1^	20 traps	X	X	X	X	X	X	X	X	X	X	X	X	X
Treatment^2^	House							X	X					
***Observations***														
Behaviour (day)	6 points* (2 cages/point)	X	X	X	X	X	X	X	X	X	X	X	X	X
Behaviour (night)	2 points* (2 cages/point)	X	X	X	X	X	X	X	X	X	X	X	X	X
Health	100 birds	X	X	X	X	X	X	X	X	X	X	X	X	X
***Samplings***														
Blood	50 birds (from 10 cages)						X		X					X
Feathers	25 birds (of 50 blood-sampled)						X							X
Production	House	X	X	X	X	X	X	X	X	X	X	X	X	X

*Points from which observations were made;

^1^Removed 2 days after placement;

^2^First fluralaner administration, 2 or 3 days before trap placement (Weeks 0 and 1), 3 or 4 days before behavioural observations (Weeks 0 and 1), 1 day before blood sampling (Week 1)

#### Daytime observations

Before beginning any daytime behavioural observation, the investigator would remain stationary in front of each cage for 10 minutes, after which bird behaviours would be recorded over a 30-minute period between 9 am and 2 pm. Six observational points were randomly selected throughout the building, focusing on the perch area of two adjacent cages. Behaviours were counted by focusing on a subset of no more than 12 hens, with observations recorded on a group, rather than on any individual bird [21]. On each day of assessment, the investigator would begin observations at 9 am, starting at the same point (Point 1) on each day, changing every 30 minutes to the next observational point and so on until reaching the final point (Point 6). The same cages were used for each observational period.

#### Night-time video observations

Two infrared equipped cameras were installed on two of the previous observation points, focusing on the perch area of two adjacent cages per observation point, the same cages for each observational period. Recordings started three hours after the onset of darkness (at midnight) when mites are most active.

Behavioural observations covered a 30-minute period, combining scan samplings and continuous behaviour recordings of selected variables (Table 2) [14, 22–24]. Specific active behaviours were recorded at group level through samplings for one minute every two minutes, meaning that 15 continuous behaviour recordings of 1-minute duration were recorded over a 30-minute period. For each 1-minute observation, the number of hen behaviours observed in the perch area was counted. Each behavioural category was considered as being mutually exclusive and was measured as the number of times the behaviour appeared in the group of hens observed. Any behaviour repeated by the same hen within 5 seconds of the previous observation of that behaviour was counted as one bout. The data were further expressed as incidence/bird/15 min-period. General activity was assessed through scan sampling every two minutes and expressed as the percentage of active hens in proportion to the total number of hens observed (active and resting). A total of 39h daytime and 13h night-time observations were made. All behavioural observations were carried out by the same investigator from the Universitat Autonoma de Barcelona.

**Table 2:** Behavioural activity assessment categories.

Behaviour	Definition
**One-minute continuous sampling of behaviours**	
**Body shaking**	Shaking while standing, with the whole body in motion
**Vertical wing shaking**	Lying or sitting: fluffed feathers, fast vertical wing movements
**Head scratching**	Scratching head with a foot
**Head shaking**	Head turning quickly from side to side, covering an angle of approximately 180 °, keeping the head above the shoulders
**Preening**	Preening own plumage with the beak
**Nipping at neck feathers**	Approaching and pecking frontally and from below
**Gentle feather pecking**	Gentle pecks to the tips of the feathers of another bird without breaking or removing feathers; often ignored by the recipient
**Severe feather pecking**	Forceful pecks/pulling of feathers (frequently eaten)—results in feather loss, especially on back, vent and tail areas. Victims often initially move away, squawk or confront the pecker
**Aggression**	Pecks directed at the head of another bird, or threats leading to an avoidance reaction of the recipient
**Scan sampling (activity levels)**	
**Resting**	Dozing or sleeping with no apparent movement [21]
**Active animals**	Active, not in a dozing or sleeping position

#### Physiological parameters

Prior to beginning the study, 5 birds per cage from 10 cages were randomly selected and identified by leg rings. To avoid any interference of the sampling on bird behaviour, the cages sampled for physiological parameters were different from those recorded for behavioural observations. Blood samples were obtained from these birds one week prior to the first fluralaner administration (Week -6) and at 1 and 6 weeks following that administration. As blood sampling procedure can greatly influence stress hormone levels, particular care was taken when catching and manipulating the birds [25]. Three weeks prior to the start of the experiment the birds were habituated to the presence of the observers and manipulation. When the experiment started, the birds did not show any fear response when the observer opened the cages, touched and caught them gently. Blood was collected by venepuncture from the wing vein into a heparin tube and into a plain tube (1 ml per tube).

Red blood cells, haematocrit, haemoglobin concentration, mean corpuscular volume, mean corpuscular haemoglobin and mean corpuscular haemoglobin concentration were measured using an automated hematology analyzer (ADVIA 120 Hematology System, Siemens Healthineers, Spain). White blood cells and heterophil to lymphocyte ratio were counted manually by examination of blood smears using a modification of the Wright-Giemsa stain (Diff-Quik). For each smear, 60 white blood cells were manually counted using a light microscope at 100x magnification [26]. Heparin tubes were centrifuged for 10 minutes at 3000 g to obtain plasma. Plasma concentrations of ovotransferrin (Chicken Ovotransferrin ELISA Kit, 157694 Abcam^®^, Cambridge, UK) and IgG (Chicken IgG ELISA Kit; Bethyl Laboratories, Montgomery, TX, USA) were determined using commercial enzyme immunoassay (ELISA) kits specific for chickens. Corticosterone was measured by a high sensitivity ELISA kit (Corticosterone HS (High Sensitivity) EIA, IDS^®^ Immunodiagnostic Systems, Boldon, UK) following manufacturer instructions. The intra-assay and inter-assay variability for this kit were less than 10% and the limit of detection was 0.17 ng/ml. The ELISA used for quantitative determination of adrenaline (epinephrine) (Adrenaline Research ELISA^™^) had intra-assay and inter-assay variability less than 10% and a limit of detection of 0.25 ng/ml. Total oxidant status, paraoxonase 1 (PON1) and total antioxidant capacity (TAC; measured by trolox equivalent antioxidant capacity) were analysed following published methods [27–29]. All determinations of oxidative stress biomarkers were performed in an automated biochemistry analyser (Olympus AU600 Automatic Chemistry Analyzer, Olympus Europe GmbH, Germany).

Changes to a bird’s HPA axis were estimated from corticosterone levels measured in 5 to 8 fully regrown feathers pulled from the interscapular area of 25 of the 50 birds selected for bleeding for the pre- and post-treatment study periods. Feather samples were all collected on the same day at Week -1 and Week +6 and individually stored at room temperature until analysis. In order to obtain the same range of length (30 cm) and minimum mass of 10 mg, four to six feathers were analyzed per bird. Feather corticosterone was extracted following a modified methanol-based technique and measured as for blood corticosterone [30]. All samples were analysed at the Interdisciplinary Laboratory of Clinical Analysis (Interlab-UMU, University of Murcia, Spain).

#### General health indicators

At weekly intervals, clinical observations were assessed in an additional 50 birds from the house. Observations were scored using the following scales.

- Feather score: 0 = no damage; 1 = slight; 2 = moderate; 3 = severe) for neck, breast, vent, back, wings and tail (modified [31])
- Comb pecking wounds: 0 = none; 1 = ≤ 3; 2 = > 3 [32]
- Comb colour: 0 = red; 2 = very pale comb
- Red mite visibility on the hen’s body: 0 = no mites; 1 = a few; 2 = many

General observations of flock health were completed daily by the study investigator or by a qualified and trained staff member. Any bird dying during the study that had been identified for feather or blood sampling was not replaced. Carcasses and eggs were disposed of in accordance with legal requirements, including applicable withholding periods.

#### Impact of PRM on flock performance

The impact of PRM infestations on flock performance and the effects of acaricidal treatment were assessed at weekly intervals using farm-recorded data from Week -6 to Week 6. Parameters that were assessed included weekly bird mortality rates, rate of egg laying (ratio of the number of eggs laid to the number of hens present) and egg weight.

### Statistical analysis

Statistical units were the mite traps for quantification of PRM infestation, the house for behavioural evaluations and general performance parameters, the individual bird for physiological parameters and health status evaluation. The percent reduction of PRMs was based on the number of mobile stages (larvae, both nymph stages and adults) in the quantitative traps pre- and post-treatment, calculated using the following formula:

$$\text{Efficacy}\;(\%)\;=\;100\times \;({\text{X}}_{\text{pre}}-{\;\text{X}}_{\text{post}})/{\text{X}}_{\text{pre}}$$

where X_pre_ is the arithmetic mean pre-treatment mobile stage mite count (Week -1) and X_post_ is the arithmetic mean post-treatment mobile stage mite count in Weeks 1, 2, or 6.

Pre- and post-treatment numbers were pairwise compared using a two-sided two-sample t-test at 5% threshold significance.

For behavioural parameters, the percentage of active hens was expressed in proportion to the total number of hens. The number of events was expressed as the number of bouts (number of times a behavioural element appeared) per hen within 15 minutes in proportion to the total number of active hens observed (Table 1). To investigate the phase (before or after treatment) as the main effect (α = 0.05), pre- and post-treatment behaviour observations were compared using a mixed linear model. The number of bouts (or % activity of hens) was the dependent variable, study phase (pre- or post-treatment) the first effect to be investigated, phase-observation point interaction the second, study week the repeated factor and observation point the random factor. Daytime and night-time observations were investigated separately.

The effects of elimination of infestations on bird welfare were determined by comparing Week -1 blood analysis results to those obtained in Weeks 1 and 6 using a two-sided two-sample t-test at 5% threshold significance. Pre- and post-treatment frequencies of comb colour were investigated using a two-sided *χ*^2^-test with α = 0.05. Differences in mean feather scores pre- and post-treatment were investigated using a two-sided Wilcoxon’s Rank Sum Test at 5% threshold significance.

Changes in production data (weekly mortality rate, laying rate and weekly egg weight) were analysed to guide further insights into the effects of elimination of PRM infestations. A linear regression model was applied to investigate if there were any significant changes observed with time. The slope of the regression line was compared to zero. A positive slope indicated that the parameter increased with time, whereas a negative slope indicated that the parameter decreased with time. Production data were also assessed in relation to published standard production data for ISA Brown laying birds [33].

## Results

### Mite infestation

**Fig 1:**
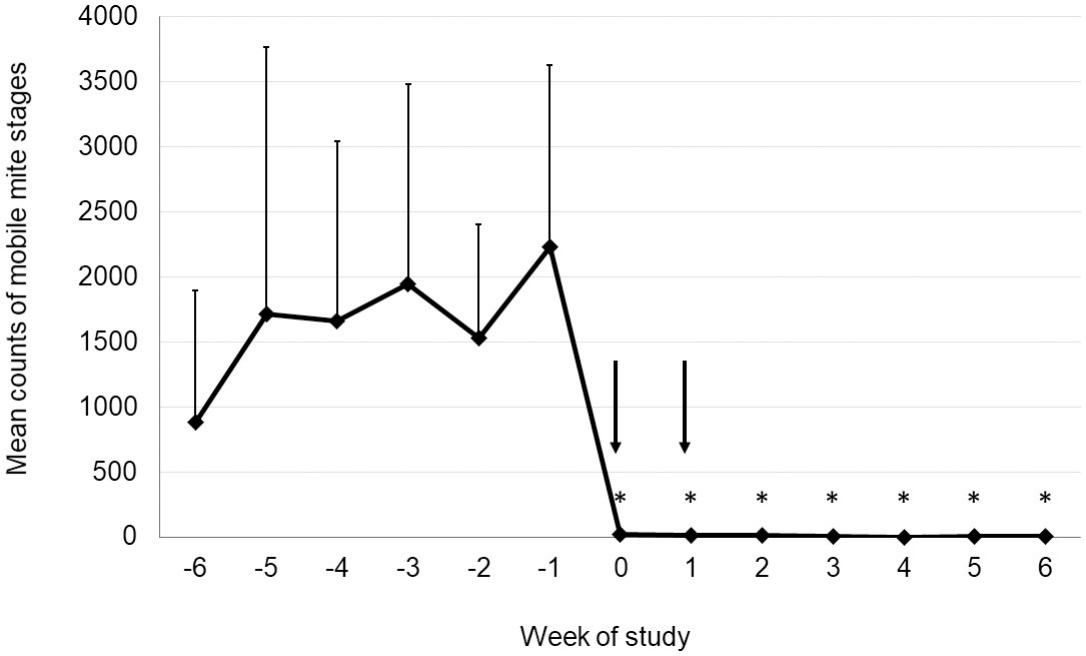
Arithmetic mean counts of mobile mites in traps. Counts in Weeks 0 through 6 differed significantly from mite counts in Week -1 (*P < 0.0001).

Counts of mobile mite stages were substantial throughout the six weeks prior to the first administration of fluralaner, reaching an arithmetic mean peak of 2,228 in each trap at Week -1 (Fig 1; S1 Dataset). In traps placed three days after the first administration of fluralaner, mite counts declined precipitously, remaining close to zero throughout the remaining six weeks of data collection. The broad-scale elimination of mites from the house environment is evident from the >99% reduction in counts at every assessment following fluralaner treatment. Mite counts in each week post-treatment differed significantly from mite counts in Week -1 (*P* <0.0001). Before treatment (Week -1) mites were observed during daytime on 6% of hens (and on the investigator). At Week 6, no mites were observed on hens at any time.

### Assessment of poultry red mite effects on infested hens

#### Behavioural observations

**Fig 2:**
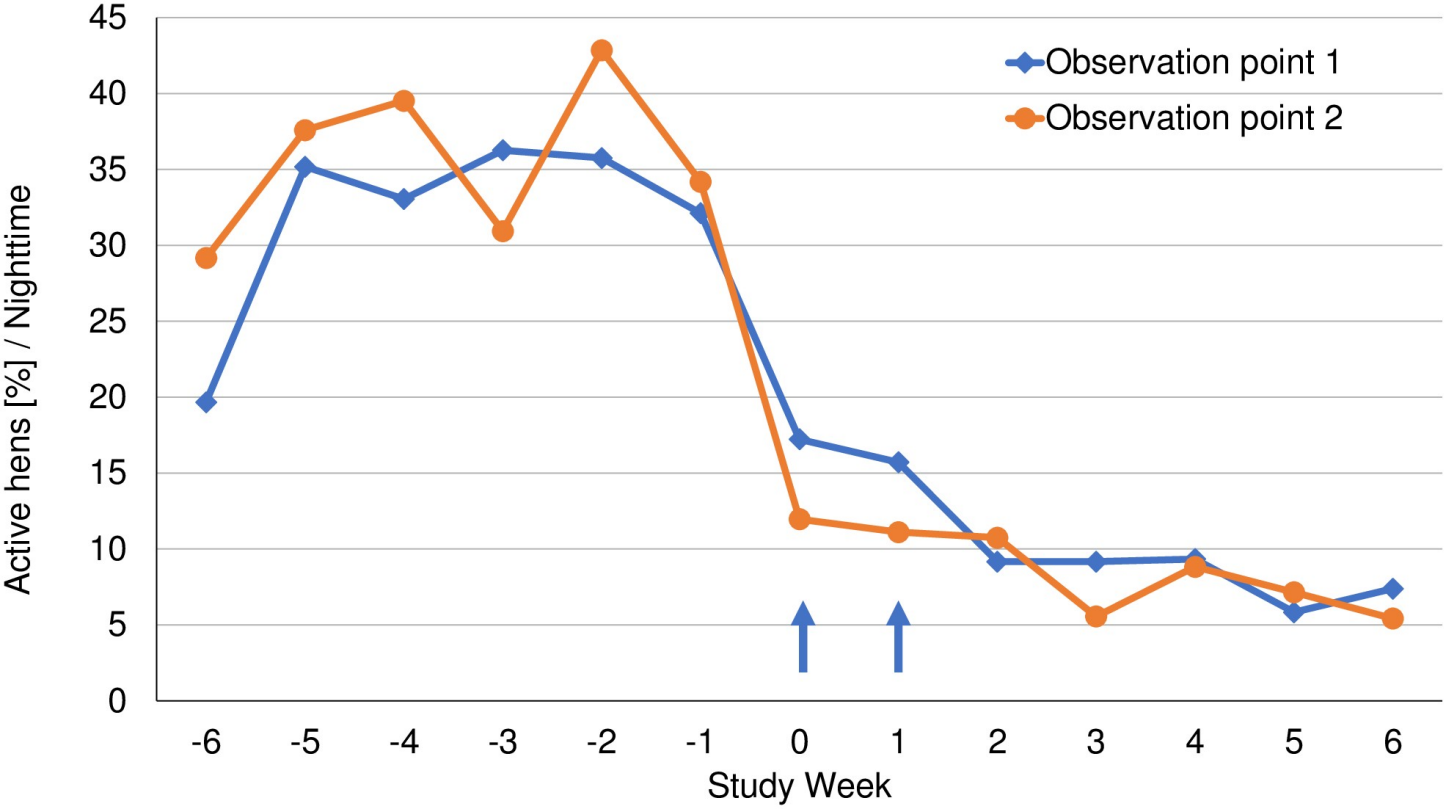
The percent of active hens observed in night-time video recordings for each observation point. Arrows indicate days of fluralaner administration (Weeks 0 and 1).

For daytime observations, more than 95% of hens were active (on average) throughout the pre- and post-treatment periods, with a significant post-treatment reduction in preening (*P* < 0.0001), head scratching (*P* < 0.0001), head shaking (*P* = 0.0389), severe feather pecking (SFP) (*P* = 0.0002) and aggression behaviours (*P* = 0.0165) (Table 3; S1 Dataset). At night-time, the percentage of active hens increased from 19.7% at Week -6 to 42.6% at Week -1. Post-treatment, the percent of active hens at night-time decreased significantly, to 5.4% and 17.2% at Weeks 1 and 6, respectively (*P* < 0.0001) (Fig 2). Night-time behaviours that were significantly reduced from pre-treatment levels were preening (*P* < 0.0001), head scratching (*P* < 0.0001), and head shaking (*P* = 0.0007) (Table 3). Vertical wing shaking was not observed at any daytime or night-time point and nipping-at-neck-feathers was insufficiently frequent for statistical modelling. There was no phase-observation point interaction detected, indicating the observations (differences between phases) are similar between observation points.

**Table 3:** Behavioural variables (least square means ± standard deviations); bouts per bird during 15 minutes observation pre- (Weeks -6 to -1) and post-treatment (Weeks 0 to +6).

	Phase	Pre-treatment	Post-treatment	*P*-value
**Preening**	Day	2.78 ± 0.21	1.97 ± 0.20	**< 0.0001**
	Night	1.33 ± 0.12	0.34 ± 0.11	**< 0.0001**
**Head scratching**	Day	0.59 ± 0.06	0.33 ± 0.06	**< 0.0001**
	Night	1.19 ± 0.08	0.23 ± 0.07	**< 0.0001**
**Head shaking**	Day	0.14 ± 0.03	0.08 ± 0.03	**0.0389**
	Night	1.54 ± 0.21	0.41 ± 0.20	**0.0007**
**Body shaking**	Day	0.12 ± 0.03	0.16 ± 0.03	0.1252
	Night	0.03 ± 0.01	0.01 ± 0.01	0.2425
**Gentle feather pecking**	Day	1.49 ± 0.14	1.57 ± 0.13	0.5063
	Night	Not observed		-
**Severe feather pecking**	Day	0.24 ± 0.04	0.10 ± 0.04	**0.0002**
	Night	Not observed		**-**
**Aggression**	Day	0.30 ± 0.04	0.20 ± 0.04	**0.0165**
	Night	Not observed		**-**

#### Physiological parameters

Significant reductions from pre-treatment were observed in mean blood corticosterone and total antioxidant levels (Table 4; S1 Dataset). The reduction in mean feather corticosterone levels, from 0.97 ng/mL prior to treatment to 0.69 ng/mL at Week 6, was not significant.

**Table 4:** Means (± standard deviations) and ranges of blood physiological parameters before and after medication with fluralaner, administered in weeks 0 and 1.

Parameter	Week -1	Week +1	Week +6	*P*-value (Week +1 vs -1)	*P*-value (Week +6 vs-1)
**Corticosterone (ng/mL)**	3.98 ± 4.48	2.75 ± 2.65	1.74 ± 2.85	0.1038	**0.0041**
**Range**	0.18–23.28	0.33–14.34	0.07–19.66		
**Adrenaline (ng/mL)**	16.99 ± 5.95	16.97 ± 6.02	16.74 ± 5.22	0.9842	0.8260
**Range**	4.7–36.0	1.1–32.6	6.5–32.2		
**IgG (mg/mL)**	2.52 ± 1.04	2.53 ± 1.08	2.32 ± 0.90	0.9763	0.3056
**Range**	0.2–5.9	0.4–5.4	0.1–4.1		
**Ovotransferrin (mg/mL)**	0.24 ± 0.13	0.22 ± 0.13	0.23 ± 0.14	0.4001	0.6436
**Range**	0.1–0.6	0.0–0.6	0.0–0.6		
**PON 1 (IU/mL)**	0.50 ± 0.06	0.51 ± 0.04	0.52 ± 0.05	0.6043	0.1263
**Range**	0.3–0.7	0.4–0.6	0.4–0.7		
**TAC (mmol/L)**	0.57 ± 0.20	0.62 ± 0.15	0.53 ± 0.16	0.1129	0.2808
**Range**	0.0–0.9	0.0–0.9	0.1–0.8		
**TOS (μmol/L)**	12.60 ± 2.23	14.72 ± 2.31	10.20 ± 2.87	**<0.0001**	**<0.0001**
**Range**	6.2–16.1	6.6–18.2	5.3–16.5		

PON 1 Serum paraoxonase; TAC Total antioxidant capacity; TOS Total oxidant status

Statistically significant pre- to post-treatment increases were found in haemoglobin (Weeks 1 and 6) and mean corpuscular haemoglobin at Week 6 (Table 5) in comparison to Week -1 (*P* < 0.0001). The reduction in H/L ratio was statistically significant between Week -1 compared to Weeks 1 (*P* = 0.0009) and 6 (*P* < 0.0001). There were no other significant changes in both blood cell counts and physiological parameters.

**Table 5:** Means (± standard deviations) and ranges of complete blood count parameters before and after acaricidal medication with fluralaner administrations in weeks 0 and 1.

Parameter	Week -1	Week 1	Week 6	*P*-value (Week +1 vs. -1)	*P*-value (Week +6 vs. -1)
**Erythrocytes (10E6/μL)**	2.1 ± 0.2	2.4 ± 0.2	2.1 ± 0.2	**<0.0001**	0.7183
**Range**	1.5–2.6	1.7–3.0	1.8–2.7		
**Haemoglobin (g/dL)**	7.0 ± 0.8	8.0 ± 0.8	7.8 ± 0.6	**<0.0001**	**<0.0001**
**Range**	5.1–9.4	5.4–9.8	6.4–9.7		
**Haematocrit (%)**	16.5 ± 2.2	17.9 ± 2.6	16.0 ± 1.8	**0.0052**	0.2337
**Range**	10.3–20.5	11.6–23.4	12.5–21.1		
**MCV (fl)**	77.2 ± 6.8	75.7 ± 7.7	75.4 ± 6.9	0.3501	0.2186
**Range**	51.4–86.8	51.9–87.8	56.5–91.0		
**MCH (pg)**	32.9 ± 2.7	33.9 ± 2.4	36.9 ± 1.4	0.0593	**<0.0001**
**Range**	26.0–39.9	29.2–39.6	33.1–39.8		
**MCHC (g/dL)**	39.5 ± 3.0	39.4 ± 2.3	39.3 ± 2.5	0.9071	0.7760
**Range**	34.5–46.0	36.1–43.8	33.2–44.4		
**Heterophils**	3.6 ± 2.1	2.5 ± 1.1	0.9 ± 1.0	**0.0007**	**<0.0001**
**Range**	0.5–11.0	0.5–5.5	0.2–5.8		
**Lymphocytes**	7.5 ± 2.1	8.3 ± 1.7	9.5 ± 1.5	**0.0461**	**<0.0001**
**Range**	4.6–15.1	4.5–12.1	6.8–13.3		
**Leukocytes (10**^**E**^**3/μL)**	11.1 ± 1.7	10.8 ± 1.6	10.4 ± 1.7	0.2694	**0.0434**
**Range**	8.0–16.8	8.0–14.8	8.0–15.2		
**H:L**	0.6 ± 0.5	0.3 ± 0.2	0.1 ± 0.1	**0.0009**	**<0.0001**
**Range**	0.1–2.2	0.1–1.2	0.0–0.6		

MCH Mean corpuscular haemoglobin; MCHC Mean corpuscular haemoglobin concentration;

MCV Mean corpuscular volume; H:L Heterophil/lymphocyte ratio

#### Bird health

No adverse events associated with the treatment were observed. The number of birds with comb wounds increased slightly pre-treatment, between Week -6 (3%) and Week -1 (6%) (S1 Dataset). At the final observation (Week 6) there were no comb wounds observed in any of the monitored100 birds, and the pre- to post-treatment difference in comb wound scores was statistically significant (*P* = 0.0127). The post-treatment improvement in comb colour between Week -1 (very pale in 21.6% of birds) and Week 6 (very pale in 3.0%) was statistically significant (*P* <0.0001).

In the pre-treatment period there was only slight evidence of feather damage on breast, vent, wings and back. However, during Week -1, there was slight to moderate feather damage in the neck region in 34% of birds, increasing to 82% of birds during Week 6. Slight feather damage in the tail area was reported during Week -1 in 48.5% of birds, and during Week 6 in 58.6% of birds. The comparison of pre- and post-treatment values (Week -1 compared with Week 6) revealed a significant increase in neck feather scores (*P* < 0.0001), and no significant differences for breast, vent back, wings and tail feather scores (*P* > 0.05).

#### Production parameters

Following the fluralaner treatment (which includes two separate administrations), hen weekly mortality rate decreased with time (slope of the linear regression curve -0.00565, *P* = 0.0169), declining from 0.0474% to 0.1186% before treatment to 0.0159% to 0.1030% after treatment (S1 Dataset). The published ISA Brown standard mortality rates for birds of the same age as study birds (29 to 41 weeks) range between 0% and 0.1% with an average of 0.0846% [33]. That reported standard mortality rate does not change significantly with time (slope of the regression curve +0.00110, *P* = 0.7109).

**Fig 3:**
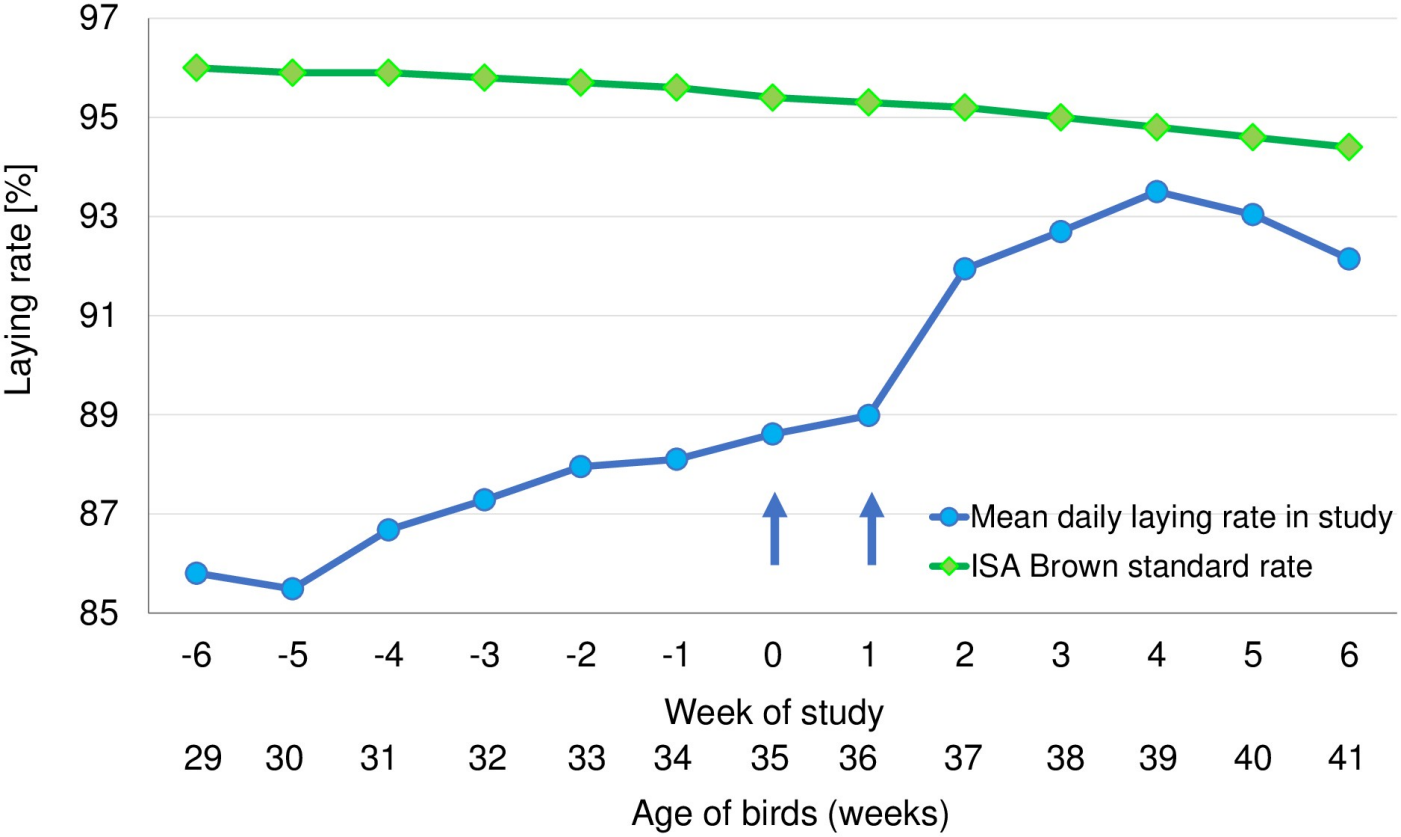
Weekly mean of daily egg laying rate of study hens before and after elimination of poultry red mites following acaricidal treatment and industry standard mean laying rates. Arrows indicate days of fluralaner administration (Weeks 0 and 1).

The increase in egg laying rates ranged between 85.5% and 88.1% pre-treatment, 88.6% and 93.5% post-treatment. The laying rate increased with time (slope of the linear regression curve +0.70436, *P* < 0.0001), whereas the published ISA Brown standard laying rates for birds in the same age range as study birds (between 29 and 41 weeks) show a decline (slope of the linear regression curve -0.13297, *P* < 0.0001). Throughout the study egg-laying rate was lower than that of the ISA Brown industry standard, although following acaricidal treatment the difference between the study results and the industry standard declined (Fig 3).

Weekly mean egg weights ranged between 59.5 g and 62.8g pre-treatment (mean 61.1 g) and between 63.7 g and 66.2 g post-treatment (65.3 g) (S1 Dataset). Egg weights increased significantly with time. The slope of the linear regression curve was +0.61319 (*P* < 0.0001), indicating a mean increase of approximately 0.6 g per week and per egg. The published ISA Brown standard egg weights for birds between 29 and 41 weeks of age also increase, but with a slope of +0.18187 (approx. 0.2 g per week).

## Discussion

The objective of the study was to evaluate changes in welfare indicators following acaricidal treatment of hens in a PRM-infested poultry unit. Under the commercial poultry conditions of this study, it was not possible to include a control group, for both ethical and practical reasons. The ethical reasons relate to worker safety and to the potential impact of PRM infestation on hen welfare. The practical reasons involve the need to treat all units of a commercial farm to avoid cross contamination between untreated and treated birds, or between treatment groups if a positive control is involved. Thus, the situation-imposed absence of a contemporary control group introduces a potentially confounding factor of age-related and environmental factors that may have influenced study findings. However, the comparison of production results with the industry standard for ISA Brown hens helps validate conclusions linking mite elimination to the observed productivity changes, including reduced hen mortality and a recovery in egg production. Moreover, before and after comparisons were made for all the studied indicators. Those productivity changes can be attributed to the improvements in bird welfare and health resulting from the near-complete elimination (>99%) of PRM infestations. Two recent reports described egg production increase after PRM elimination using fluralaner [16, 34].

The level of ectoparasite infestation is an important factor governing the amount of time birds spend in grooming behaviours, such as preening with the beak and head scratching with the feet [35–39]. In the current study, the reduced frequency of those behaviours is a likely effect of mite elimination. As preening is an energetically expensive activity that can increase metabolic rates by as much as 200%, the significant reduction of grooming behaviour seen in the study may explain at least part of the improved post-treatment productivity observed herein [40, 41].

Night-time head shaking behaviour was described as being an “alerting response” and anticipation of a negative event, and indicates that hen welfare is compromised by being in a less-preferred environment [42–45]. The reduction in head shaking following treatment for PRM indicates that infestations contribute substantially to this behaviour, and that reduction of head shaking following treatment provides further evidence of the overall effect of infestations on bird welfare.

The significant reduction in daytime feather pecking is consistent with earlier studies. Infestation with *D*. *gallinae* was identified as an important risk factor for SFP which was shown to be more frequent under conditions of high PRM challenge [2, 13]. While it is recognized that the PRM favours host-seeking during night-time, the daytime observations of infested hens were recorded previously [46], and in the experience of the investigators of the current study is more likely to be observed when environmental mite numbers are very high, as they were in the current study, prior to the first administration of fluralaner.

Although motivations for aggressive bird behaviours are not identical with those of feather pecking, both behaviours can be triggered by chronic pain or frustration [47–50]. These behaviours were reduced after the acaricidal treatment, with an associated significant reduction in comb wounds. While feather damage to the neck can be attributed to aggressive behaviours such as SFP, other causative factors include molting and abrasion against the housing system [47]. We believe that the latter factor was the cause of a post-treatment increased percentage of hens with feather damage in the neck area, arising from rubbing against the wire of the cages while feeding.

In the pre-treatment period, active night-time behaviours were recorded from approximately 40% of observations. After the fluralaner treatment active behaviours declined to less than 10% of pre-treatment night-time observations, similar to levels that were regarded as being normal for night-time activity [22]. Several physiological processes such as energy conservation and tissue restoration were indicated to take place during rest, which may be vital for the animal [51]. Disturbance of rest caused by high PRM infestation intensity substantially interferes with animal welfare and may be linked to reduced performance.

Two manifestations of bird stress, increases in blood corticosterone levels and H/L ratios, were significantly reduced following the elimination of the mite challenge, continuing to drop through the 6-week post-treatment period [14, 52]. Infestation with PRM seems to be a stressor that activates the HPA axis with the consequent release of corticosterone into blood, accounting for the increased blood levels during infestations, and measuring HPA axis activity is the standard approach to the study of stress and welfare in farm animals [25, 53]. Feather corticosterone concentration provides an assessment of corticosterone production over the period of feather growth, and as such is a unique integrated measure of avian glucocorticoid physiology for monitoring axis changes over relatively long time frames [14, 54, 55]. In the present study, the change in feather corticosterone concentrations during the 6-week pre-treatment and 6-week post-treatment periods was not significant. Direct correlations between plasma corticosterone and feather corticosterone may not always be expected and there is a need for caution if using this measurement as a proxy for plasma measures [55].

Also, in stressful situations, there is an increase in heterophils and a decrease in lymphocytes, giving an increase in the H:L ratio [52]. The post-treatment decreases we observed in these stress markers indicate that there was a reduction in PRM-induced physiological stress.

Recent studies in animals showed that health disorders such as idiopathic inflammatory bowel disease [56], pyometra [57], parasitic diseases such as babesiosis [58], and mammary tumours [59] can cause oxidative stress. Indicators of oxidative stress include increases in total oxidant status (TOS) and TAC. In the present study, serum TOS increased one week after treatment. However, at six weeks post-treatment reductions from pre-treatment levels were significant, demonstrating lower levels of oxidants and an improvement in the oxidative state. However, consistent with an earlier work in dogs treated for leishmaniosis, the TAC did not change following treatment [28]. Further studies should be undertaken to elucidate the reason for the changes in stress oxidative biomarkers after treatment for PRM infestation that we observed.

Serum PON-1 is an antioxidant enzyme which protects lipoproteins from peroxidation, among other functions, and is considered to be a negative acute phase protein (APP) [60]. Ovotransferrin also is typically specified as a negative APP and its protective role in the innate immune system is through sequestration of ferric ions to prevent parasites and pathogens from using nutrients [61]. No inflammatory changes measured through APP levels were observed in the present study. The absence of high levels of APPs when hens were severely infested by red mites could be due to the inhibition of the inflammatory response by high levels of corticosteroids which were shown to reduce inflammatory response in dogs [62].

Blood count parameters indicated that haemoglobin levels and mean corpuscular haemoglobin increased following mite elimination. The first clinical sign observed in PRM-infested animals is sub-acute anaemia due to repeated mite bites [4]. A PRM-infested laying hen can lose more than 3% of its blood volume every night [6]. In extreme cases, *D*. *gallinae* burdens may be so heavy that hens die from severe anaemia [63, 64]. In the current study, the high percentage of hens with very pale comb colour prior to treatment was likely due to the severe PRM challenge, and the improvement in comb colour following mite elimination a reflection of improved blood parameters.

## Conclusion

The objective of this study was to evaluate welfare changes observed in ISA Brown laying hens on a commercial building following the elimination of PRM by drinking water treatment with a highly effective acaricide. The >99% reduction of mite challenge was accompanied by improvements in indicators of hen welfare, including reductions in blood corticosterone levels and behavioural variables of night-time activity, preening, head scratching and head shaking. During daytime there were post-treatment reductions in SFP and aggressions. Health improvements were evidenced by reductions in H:L ratio and TOS and a recovery in haemoglobin and mean corpuscular haemoglobin levels. Further, following the elimination of mite challenge, egg production recovered, and hen mortality decreased. Allowing for the absence of a contemporary control group, the findings indicate that infestation with PRM impairs hen welfare which can be enhanced when hens are freed from PRM challenge.

## Supporting information

S1 Dataset Source data for mite counts, behaviour parameter observations, poultry health and productivity parameters. (XLSX)

## Abbreviations

APP: Acute phase protein
CVMP: Committee for Medicinal Products for Veterinary Use
GCP: Good Clinical Practice
HPA: Hypothalamic-pituitary-adrenal
PON 1: Paraoxonase 1
PRM: Poultry red mite
TAC: Serum total antioxidant capacity
TOS: Total oxidant status
VICH: International cooperation on harmonization of technical requirements for registration of veterinary medicinal products
X_post_: mean post-treatment mobile stage mite count
X_pre_: mean pre-treatment mobile stage mite count (Week -1)
